# Effects of food‐borne nanomaterials on gastrointestinal tissues and microbiota

**DOI:** 10.1002/wnan.1481

**Published:** 2017-05-26

**Authors:** Hans Bouwmeester, Meike van der Zande, Mark A. Jepson

**Affiliations:** ^1^ Division of Toxicology Wageningen University and Research Wageningen The Netherlands; ^2^ RIKILT ‐ Wageningen University and Research Wageningen The Netherlands; ^3^ School of Biochemistry University of Bristol Bristol UK

## Abstract

Ingestion of engineered nanomaterials is inevitable due to their addition to food and prevalence in food packaging and domestic products such as toothpaste and sun cream. In the absence of robust dosimetry and particokinetic data, it is currently challenging to accurately assess the potential toxicity of food‐borne nanomaterials. Herein, we review current understanding of gastrointestinal uptake mechanisms, consider some data on the potential for toxicity of the most commonly encountered classes of food‐borne nanomaterials (including TiO_2_, SiO_2__,_ ZnO, and Ag nanoparticles), and discuss the potential impact of the luminal environment on nanoparticle properties and toxicity. Much of our current understanding of gastrointestinal nanotoxicology is derived from increasingly sophisticated epithelial models that augment in vivo studies. In addition to considering the direct effects of food‐borne nanomaterials on gastrointestinal tissues, including the potential role of chronic nanoparticle exposure in development of inflammatory diseases, we also discuss the potential for food‐borne nanomaterials to disturb the normal balance of microbiota within the gastrointestinal tract. The latter possibility warrants close attention given the increasing awareness of the critical role of microbiota in human health and the known impact of some food‐borne nanomaterials on bacterial viability. *WIREs Nanomed Nanobiotechnol* 2018, 10:e1481. doi: 10.1002/wnan.1481

This article is categorized under:

1 Toxicology and Regulatory Issues in Nanomedicine > Toxicology of Nanomaterials

## INTRODUCTION

As nanotechnology expands there is an increasing need to assess the potential consequences of exposure to nanomaterials. The oral route of exposure to nanomaterials is an important consideration because of their deliberate addition to food, their widespread use in food packaging and other domestic products, and potential for inadvertent ingestion from environmental contamination.^1^ Many (nano)materials, including silicon dioxide (SiO_2_), titanium dioxide (TiO_2_), silver (Ag), and zinc oxide (ZnO) nanoparticles (NPs) are currently added to food,^2^, ^3^, ^4^ which, together with those nanomaterials in products such as toothpaste, cosmetics, and sun cream, have a clear potential for ingestion by a large proportion of the population. Consideration of oral exposure to nanomaterials also overlaps with the inhalation route because a considerable proportion of inhaled material reaches the gastrointestinal tract (GIT) following clearance from the respiratory tract. In recent years there has been much progress in our mechanistic understanding of nanotoxicology and increased awareness of the behavior and interaction of nanomaterials in the GIT. Nevertheless, there is no clear consensus regarding the potential impact of these materials within the GIT or the extent to which they may be translocated to the circulation because the exposure to nanomaterials is still poorly quantified and few studies have estimated the daily exposure to nanomaterials via food.^4^, ^5^, ^6^

Here, we will review the routes for GIT uptake and transport of nanomaterials. *In vivo* assessment of gastrointestinal uptake and distribution of nanomaterials, including food‐related studies, has confirmed uptake and tissue distribution,^7^, ^8^, ^9^, ^10^ but has not conclusively demonstrated significant risk due to low uptake rates and uncertainties over exposure levels.

We will summarize current knowledge of the mechanisms of toxicity of nanomaterials in the gut, and highlight areas where information is lacking. Clearly, a thorough assessment of specific nanomaterials is beyond our scope and the reader is kindly referred to excellent recent reviews. We will include a consideration of *in vitro* models and their application for mechanistic nanotoxicology studies. The more elegant studies exploit sophisticated models that seek to mimic the complex morphology, environment, and cellular interactions that characterize the gastrointestinal epithelium. Although acute toxicity of nanomaterials to epithelial cells has been suggested from both *in vitro* and *in vivo* studies there is little evidence that this is a realistic risk due to low doses likely to be ingested. Similarly there are few reports of causal links between nanomaterial ingestion and gut pathology, with the notable exception of the potential role of TiO_2_ nanomaterials in development of colitis and cancer.^11^, ^12^, ^13^, ^14^ Concerns remain over potential chronic effects of nanomaterials on gut mucosa or the microbiota resident in the gut lumen, especially because the delicate balance between microorganisms and their host is increasingly identified as a critical factor in gastrointestinal and metabolic diseases.

## CONDITIONS IN THE GASTROINTESTINAL TRACT

After nanomaterials enter the organism via the oral route they are subjected to conditions that are very different from those encountered via other exposure routes.^15^ Perhaps most significantly, the extremely low pH of the stomach and the high ionic strength in the stomach and intestine will critically affect nanomaterial properties,^2^, ^16^, ^17^, ^18^, ^19^ potentially yielding products with differing toxicity profiles. Further, pH changes in the small intestine, mucus, and resident microbiota in the GIT lumen add to the complexity of the environment encountered by nanomaterials within the GIT and affect their potential toxicity.

### Nanomaterial Interactions With the Gastrointestinal Environment

Physicochemical properties and aggregation of nanomaterial will be affected by extreme pH and ionic shifts encountered during gut transit, by co‐ingested material such as food matrices, and by the proteins, mucus, and bile acids secreted within the gut.^20^ Attempts to model the influence of the gut environment on nanomaterials include the use of an *in vitro* digestion model to assess the fate of 60‐nm silver nanomaterials, and nanometer‐sized silica in the gut.^2^, ^16^ These studies showed that the GIT environment as well as the presence and composition of a food matrix affect the nanomaterial properties during transit before it is available for uptake in the small intestine.^21^, ^22^

### Nanomaterial Interactions With the Intestinal Microbiome

One underexplored area of relevance is the potential effect of nanomaterials on the normal bacterial microflora, which is now known to play key roles in GIT development and regulation of inflammation.^23^, ^24^ As reviewed by Fröhlich and Fröhlich,^25^ it is important to realize the cellular differences between eukaryotic and prokaryotic cells. Bacteria lack protective membranes around their DNA (as they do not have a nucleus) and lack specific active uptake mechanisms, such as endocytosis as present in mammalian cells. In addition, they possess a cell wall that might pose a barrier for nanomaterials. Thus, for nanomaterials to cause an effect to bacteria, the cell wall needs to be destroyed. Many of the antimicrobial effects of nanomaterials therefore have been attributed to ions dissociated from nanomaterials.^25^ A limited number of studies on the interaction of nanomaterials with the microbiome is available, most of them in rodents. Some case studies will be highlighted here, concluded by observed trends and some experimental considerations. *In vitro* studies employing single bacterial strains were not included.

In mice, orally exposed to 55‐nm Ag nanomaterials (0, 46, 460, or 4600 ppb) for 28 days,^26^ no overall toxicity was recorded. However, the exposure disturbed bacterial evenness in the colon, a condition that has often been related to pathological conditions. Ag NPs increased the ratio between Firmicutes and Bacteroidetes phyla. Strikingly no effects were observed upon exposure to aged sulfated Ag nanomaterials. The authors speculated that the changes in corona, rapid gastric dissolution, and subsequent precipitation as AgCl and Ag_2_S^16^, ^27^ might affect the antimicrobial effect. Comparable effects were found in ileal samples following 13‐week exposure to Ag NPs (20 and 110 nm with Polyvinylpyrrolidone (PVP) and citrate coatings at 10 mg/kg body weight/day).^28^ However, in an earlier study using the same materials and dosing for 28 days,^29^ no effects of Ag NPs on the intestinal cecal microbiota were reported. This disparity may reflect differences in microbiota within the GIT and highlight the importance of fecal sampling area in such studies. Chitosan nanomaterials loaded with copper sulfate orally exposed to rats for 21 days affected the cecal microbiota composition and increased butyrate production. Butyrate serves as a key energy source^30^ and as a critical mediator in inflammatory responses in intestinal cells.^31^, ^32^ A 35‐day study in mice, orally exposed to particulate matter (PM10) at 10–13 mg/g/day per mouse,^33^ showed an altered microbial composition in the colon, an increased proinflammatory cytokine expression, and decreased butyrate concentrations.^33^ Lastly, human microxbial donor extracts were incubated with CeO_2_, TiO_2_, and ZnO nanomaterials for five subsequent days in a model colon reactor.^34^ While this study did not monitor the changes in the ecosystem of microbiota, the authors reported significant phenotypic changes including in the production of extracellular polymeric substance and short‐chain fatty acids upon incubation with the nanomaterials (most prominently for TiO_2_) and a decreased butyrate production upon exposure to CeO_2_ NPs.^34^

In summary, nanomaterials can affect the microbial composition and its butyrate homeostasis, but the underlying mechanisms and the toxicological implications are still unclear. It is important to note that there are differences between the human and rodent microbiome although detailed comparison is not yet possible in the absence of full annotation of microbiome species in gene databases. Furthermore, nanomaterial–microbiome interactions appear to be sampling area dependent. Generally, there is a bias to examine the microbial composition in the large intestine, whereas the composition can be very different in another area. For instance, the microbial composition in the human small intestine was reported to be far less complex than that of the large intestine.^35^ Finally, a crucial aspect for *in vitro* studies is to take care to incorporate the changing physiochemical properties of nanomaterials (i.e., aging of nanomaterials) during transit of the GIT in the study design.

## NANOMATERIAL UPTAKE IN THE GASTROINTESTINAL TRACT

Mechanistic information regarding nanomaterial uptake routes in the GIT has been almost exclusively derived from cell culture models that are both amenable to experimental manipulation and more readily interpretable. A variety of *in vitro* GIT models have been developed and used for nanomaterial uptake studies.^10^, ^36^

The cellular composition of the GIT epithelium is a key determinant of nanomaterial interactions. The small intestine is mostly lined by enterocytes, columnar epithelial cells with a dense microvillus brush border, and sealed by tight junctions (TJs) that prevent passage of most materials. Interspersed between enterocytes are goblet cells that secrete mucus into the gut lumen and thereby provide an additional barrier to diffusion of particulates toward the epithelium.^37^ The extent to which mucus blocks particle diffusion depends on particle size and surface properties, with smaller particles penetrating more and positively charged particles being mucoadhesive.^37^ The apical membrane of enterocytes is also covered with a complex glycocalyx, composed of glycosylated proteins within the membrane, which forms a size‐selective barrier to the interaction of particulate material with surface molecules.^38^, ^39^ The ability of the glycocalyx to block nanoparticle translocation is determined by its thickness, density, negative charge, and renewal characteristics.^40^, ^41^, ^42^ Peyer's patches and other gut‐associated lymphoid tissues accumulate particulate material due to the presence of antigen‐sampling M cells at these sites, which exhibit a remarkable capacity to transport material, including proteins, inert particles, viruses, and bacteria, and deliver them to the underlying lymphoid cells.^43^, ^44^

As an *in vitro* epithelial barrier model, fully differentiated Caco‐2 cells (human epithelial colorectal adenocarcinoma cells) are commonly used. It has been reported that diverse nanomaterials, including TiO_2_, SiO_2_, CeO_2_, and Ag NPs, are transported across this epithelium,^20^, ^45^, ^46^, ^47^, ^48^, ^49^, ^50^ albeit sometimes negligible for TiO_2_.^51^ Interestingly, the *in vitro* uptake in differentiated Caco‐2 cells of 5‐nm nano Fe(III) (iron hydroxide adipate tartrate) nanomaterials correlated well with human absorption.^52^

There is no clear consensus as to which uptake route—transcellular or paracellular—is more important and this almost certainly depends on the specific properties of individual nanomaterials. As epithelial cells form a tight dense monolayer, it is often assumed that transcellular routes predominate. Experiments with cultured GIT epithelial cells^47^, ^53^ and *in vivo* mouse studies^54^ have demonstrated the capacity of enterocytes to internalize nanomaterials. It is likely that particles in the nanosize range are internalized through clathrin‐ and/or caveoli‐dependent endocytosis, which operates in polarized epithelia,^54^, ^55^ while uptake of larger particles (>100 nm) occurs mainly by phagocytosis and micropinocytosis, which do not take place to a significant extent in fully differentiated GIT epithelia (with the exception of M cells).

The best characterized route for transcellular particle translocation is that through specialized antigen‐transporting M cells in Peyer's patches and other lymphoid tissues in the GIT. M cells are known to transport a broad range of particulate materials including inert particles^43^, ^56^, ^57^ and pathogens.^44^, ^58^ Recent work shows that the presence of M cells, induced by Raji cells, in a Caco‐2 cell model increased the translocation of solid lipid nanoparticles (ranging in size from 50 to 70 nm with different surface modifications).^59^ However, transport through M cells does not necessarily mean that the nanomaterials reach the bloodstream as M cells are closely associated with immune cells. A study describes that orally administered glucan and poly(lactic‐*co*‐glycolic acid) NPs in mice were transported through M cells and subsequently endocytosed by dendritic cells (DCs) in the Peyer's patches, thereby not reaching the bloodstream.^60^ The close connection to the immune system also indicates that the intestinal immune homeostasis may be influenced by nanomaterials. One study describes spontaneous formation of amorphous magnesium‐substituted calcium phosphate nanoparticles from calcium and phosphate ions that are naturally secreted into the lumen of the distal small intestine. These particles trap soluble macromolecules, such as bacterial peptidoglycan and orally fed protein antigens, and enter the Peyer's patches via M cells.^61^

Despite their well‐established transcytotic capacity, M cells are scarce and so the less efficient routes across the bulk of the GIT epithelium may be quantitatively more important for nanomaterial uptake. Indeed, significant uptake of particulate material has been reported in rat GIT, with no preference for Peyer's patches compared with villi.^37^, ^62^ Earlier studies reported that while larger particles are preferentially taken up by rat Peyer's patches, uptake by normal villi was significant and became more so as particle sizes decreased to 100 nm.^63^, ^64^ These data suggest that nanosized particles may access additional uptake routes to those available for larger particles and support the concept that lower efficiency of nanomaterial uptake in villus epithelium might be offset by its vastly greater surface area compared with the specialized M cells.

TJs between GIT epithelial cells limit paracellular transport and are essential for normal barrier and transport functions. Nevertheless, some studies have reported that nanomaterials of 1–2 nm might penetrate TJs to access the paracellular route across the epithelial barrier.^9^ Furthermore, polycationic molecules, which can trigger reversible opening of TJs,^65^ are being investigated for their potential to increase absorption of particulate delivery vehicles.^66^ Other studies have documented transient opening of TJs of polarized Caco‐2 cell layers by polymer‐coated gold nanoparticles of 5–20 nm diameter with varying surface charge^67^ and by nano‐sized insulin carriers formed from amine‐modified polyesters.^68^ Dendrimers, which are <10‐nm nanomaterials with potential as drug carriers,^69^ are transported across Caco‐2 cell layers with concomitant loss of transepithelial electrical resistance, suggesting that paracellular transport predominates.^70^

Therefore, except when nanomaterials are small enough or have surface properties that increase TJ permeability, paracellular transport is probably not a major route for nanomaterial penetration of the healthy GIT. However, there will not be such a strict limitation on paracellular transport in areas where the epithelium is damaged, during normal cell turnover at villus tips and in pathological states where GIT translocation may be enhanced. For example, it is well known that bacterial translocation is enhanced by conditions such as trauma, inflammation, stroke, and chronic alcohol use.^71^, ^72^ This translocation is likely to be mirrored by increased uptake of inert material, a concept supported by *in vitro* studies demonstrating enhanced penetration of 2‐µm polystyrene particles across cultured Caco‐2 cells following alcohol treatment or irradiation, which enhance TJ leakage.^73^, ^74^

In addition to the now well‐established use of polarized, co‐culture, and three‐dimensional (3D) epithelial models, recent advances in miniaturization and microfluidics have resulted in development of organ‐on‐a‐chip models.^75^, ^76^, ^77^, ^78^ Such devices enable precise control of the cell microenvironment (e.g., physical and chemical parameters) and dynamic culture conditions.^79^ For example, Caco‐2 cells grown in a gut‐on‐a‐chip model integrating peristaltic motion with dual flow to mimic both lumen and plasma exhibit features more closely resembling *in vivo* GIT, including mucus production and development of microvilli.^80^

## NANOMATERIAL UPTAKE FOLLOWING INGESTION BY HUMANS

Human volunteers (*n* = 9) were orally exposed to 5 mg/kg body weight (315–620 mg per person) TiO_2_ particles (10, 70, and 1800 nm) in a single dose. There were no indications of system uptake as indicated by urine measurements 72 h postexposure. In addition, no values outside clinical ranges (whole blood erythrocytes) were observed.^81^ However, in a comparable study where seven human volunteers ingested 100 mg food‐grade TiO_2_ NPs (mean size 260 nm), TiO_2_ was observed in blood 2 h after administration, which peaked at 6 h following ingestion.^82^ This study supported earlier findings where blood samples contained increased levels of TiO_2_ after ingestion of 160‐ and 380‐nm TiO_2_ NPs.^83^ The presence of reflective particles in blood was interpreted as evidence of the presence of TiO_2_ particles, but this was not confirmed by direct analysis of particle composition, for example, by single particle Inductively coupled plasma mass spectrometry (ICPMS).

## EFFECTS OF NANOMATERIALS ON GASTROINTESTINAL EPITHELIUM

It has frequently been noted that there is a relative paucity of data on the potential toxicity of food‐borne nanomaterials, which is at least in part due to shortcomings of *in vitro* models, limited information about exposure levels, and the complexity of the GIT environment.^84^, ^85^ There is accumulating evidence of (systemic) toxicity of metal (oxide) nanomaterials (e.g., copper, silver, silica, and titanium) in rodents following oral administration.^86^, ^87^, ^88^, ^89^ It is likely that ion release triggered by low gastric pH contributes to the toxicity of Cu and Ag NPs.^87^, ^88^, ^89^, ^90^ As discussed above, pH changes encountered during gastrointestinal digestion not only affect both stability and dispersal of nanomaterials, but also influence local toxicity.^91^, ^92^ This possibility has been investigated by mimicking the effects of gastric fluid *in vitro* which has, for example, revealed that acid‐mediated release of cadmium ions from CdSe quantum dots exacerbates their toxicity in GIT cells.^93^

In addition to the effects on nanomaterials of intrinsic gastrointestinal environment it is also important to consider the influence of food matrix on the physicochemical properties of nanomaterials and their potential toxicity. While most *in vitro* and *in vivo* GIT exposure studies are simplified using standardized cell culture media or animal feeds, in reality the exposure is complex, with the intestinal epithelium being exposed to combinations of food ingredients and nanomaterials. Some studies are emerging that explore these potential interactions. Co‐exposure of Ag nanomaterial (and Ag ions) with quercetin (a flavonol with antioxidant properties) suggested a reduced cytotoxicity and oxidative stress when compared with Ag NP exposure only.^93^

The well‐documented inflammatory responses induced by nanomaterials in airways raise concern that nanomaterials are potential risk factors in inflammatory bowel disease (IBD). It is known that human Peyer's patches accumulate pigmented material, including TiO_2_, from the gut lumen and that similar material is present in inflammatory aggregates of Crohn's disease patients.^94^ The hypothesis that particulates, such as TiO_2_, that are regularly ingested from toothpaste, etc. might play a role in the pathogenesis of IBD and related disorders^95^, ^96^ has recently been supported by experimental evidence. Administration of TiO_2_ NPs to rodents has been shown to induce inflammation in the small intestine,^11^ exacerbate colitis,^12^ promote colitis‐associated tumors,^13^ and induce colonic inflammation and preneoplastic lesions.^14^ These animal studies used high doses of TiO_2_ NPs (10–100 mg/kg/day) which are far in excess of the recently published estimates of average intake levels in the Dutch population of 0.55 µg/kg/day (adult) and 2.16 µg/kg/day (infant) and also greater than estimates of total TiO_2_ intake <1 mg/kg/day.^6^ Thus, further work is needed to reevaluate potential toxicity using realistic and chronic doses. It is also important to consider that both IBD and cancer are multifactorial, with some individuals being genetically predisposed to development of disease and/or exposed to varying levels of additional risk factors. This, together with the variable and chronic nature of intake of particulates adds to the challenge of risk assessment of nanoparticle ingestion.^97^

Nanomaterial toxicity is commonly investigated *in vitro* in undifferentiated Caco‐2 cells and less often in fully differentiated Caco‐2 cells. The latter better reflect the native GIT and are generally less sensitive in showing cytotoxicity^51^ or in producing cytokines in response to exogenous materials.^98^ Nevertheless, recent studies have reported adverse responses to Ag NPs^21^, ^99^, ^100^, ^101^ and ZnO NPs^98^ in differentiated Caco‐2 cells although others have attributed the effects of Ag NPs in differentiated Caco‐2 cells, including the Caco‐2/M cell co‐culture model, to Ag ions.^102^

Clearly there is a need to study the effects of prolonged nanomaterial exposure *in vitro*. Recently, some reports have been published claiming to have studied this. However, a study design where multiple generations of Caco‐2 cells are exposed to nanomaterials cannot be used sensibly for this, as this does not reflect the cell renewal at the intestinal epithelium *in vivo*. For this, novel models need to be developed, intestinal organoids, containing stem cells, might be one of the most promising *in vitro* models. Also the effects of nanomaterial exposure on models that reflect inflammatory intestinal epithelial disorders are interesting to explore. For this, human macrophages (THP‐1) and human DCs (MUTZ‐3) were embedded in a collagen scaffold and seeded on the apical side of transwell inserts. Caco‐2 cells were seeded and differentiated on top of this layer, forming a 3D model of the intestinal mucosa. Toxicity of nanomaterials (NM101 TiO_2_, NM300 Ag, Au) was evaluated in noninflamed and inflamed co‐cultures, and also compared to noninflamed Caco‐2 monocultures. The inflamed co‐cultures released higher amounts of IL‐8 compared with Caco‐2 monocultures. However, the cytotoxicity of Ag NPs was higher in Caco‐2 monocultures than in 3D co‐cultures. Ag NPs were found to be more toxic than TiO_2_ or Au NPs.^103^ Furthermore, intestinal organoids also seem very promising for the development of diseased gut models, but such models are not established yet.^104^

## DOSIMETRY, SEDIMENTATION, AND KINETICS

Extrapolation of *in vitro* studies to the *in vivo* GIT must be approached with caution because the true GIT is likely less responsive, but on the other hand prone to specific damage mechanisms that cannot be modeled *in vitro*. Meaningful interpretation and comparison of the results obtained using different *in vitro* experiments and extrapolation to *in vivo* data require reliable characterization of the nanomaterials and their aggregates, as well as matrix‐based influences on nanomaterials. This information is required to derive the nanomaterial dose in the testing system. For soluble chemicals it is reasonable to assume that the administered dose (or nominal media concentration) is proportional to the cellular dose, and thus is a good measure of the dose at the target site.^105^ However, the definition of a nanomaterial dose in an *in vitro* system is far more complicated. Nanomaterials can settle, diffuse, and aggregate differentially, which is determined by the properties of the nanomaterial itself (e.g., size, density, and surface chemistry) as well as by the solution (e.g., viscosity, density, and presence of proteins). Likewise, nanomaterial dosimetry is affected not only by the amount and time but also by the nanomaterial characteristics and the environment.^105^

In their 2007 review, Teeguarden et al.^105^ introduced the term particokinetics to incorporate processes that affect the nanomaterial target dose: processes that affect diffusion, gravitational settling, and agglomeration. Diffusional transport is less important for particulates with sizes above ~100 nm, while gravitational settling increases with particle density and the square of particle diameter. Based on experimental work with polystyrene, iron oxide, and silica nanomaterials, a computational *in vitro* sedimentation, diffusion, and dosimetry model was developed and refined.^106^, ^107^, ^108^ In these models the accurate determination of the effective density is crucial.^109^ They convincingly show that nanotoxicological studies that only rely on nominal media concentrations can result in erroneous conclusions on potency of nanomaterials, as differences in the extent and rate of transport of nanomaterials are unaccounted for.^105^ This possibility has been evaluated in subsequent experimental studies. However, Liu et al. compared 24 metal (oxide) nanomaterials, ranging in hydrodynamic sizes from 150 to 465 nm and effective densities from 1.3 to 3.2 g/mL, and concluded that the toxicity ranking for these metal (oxide) nanomaterials was similar using either the nominal media concentration or the calculated delivered dose.^110^ Likely, only for nanomaterials with smaller effective densities (i.e., closer to the cell culture medium) sedimentation is a crucial aspect to be considered. In contrast, Pal et al. studied seven low aspect ratio nanomaterials (effective densities from 1.0 to 2.3 g/mL; hydrodynamic sizes ranging from 145 to 464 nm) and showed that the *in vitro* cell death slopes expressed as deposited dose correlated better to *in vivo* lung inflammation than the same data expressed as administered dose.^111^

## CONCLUSIONS

Widespread exposure to nanomaterials via ingestion is an inevitable consequence of the expanding use of nanomaterials in food and other consumer products and is a cause for concern. However, the extent of ingestion and the potential risks this exposure imposes remains poorly defined. It is now well established that the physicochemical characteristics of nanomaterials are heavily influenced by their local microenvironment. Especially for the oral exposure route the influence of gastrointestinal conditions on nanomaterials needs to be considered.

Several *in vitro* models of the gastrointestinal epithelium have been developed, ranging from monolayers of a single cell type (often Caco‐2 cells) to more complex co‐cultures that, for example, incorporate M cells and mucus‐secreting cells. While these models aim to reproduce the complex biology of the intestinal epithelium, the design and composition of the nanomaterial exposure conditions also needs refinement to better reflect the real‐life human oral exposure. In some of the more recent studies cells have not only been exposed to pristine nanomaterials but also to so‐called aged nanomaterials. These aged nanomaterials have been either incubated in the gastrointestinal lumen or undergone artificial aging procedures. This often results in multielemental composition of the nanomaterials (i.e., AgS) that might have a different toxic potency compared with the pristine nanomaterial.

Currently, reported *in vitro* data suggest limited cellular uptake and epithelial translocation. However, most studies do not take adequate consideration of the dosimetry (and sedimentation) in their model system. Computational models (supported by experimental data) indicate that nanomaterial size and effective density strongly determine the nanomaterial availability for cellular uptake in static conditions. If the observed effects or uptake (rates) are only compared to the administered dose, erroneous conclusions might be reached. It is thus highly recommended to take the dosimetry, that is, the dose at the target site into consideration when interpreting the results. The recent innovations toward microfluidic experimental models might also improve the relevance of the exposure conditions. These experimental innovations need to be embedded in particokinetic and dynamic modeling of nanomaterials to extrapolate data from *in vitro* to *in vivo*.

Only recently have the potential effects of nanomaterials on the intestinal microbiota been studied. The limited data suggest effects of the nanomaterial (or dissociated ions) on the microbial ecosystem itself and also on the release of short‐chain fatty acids that are known to be important in the interactions with the intestinal epithelium. Clearly the nanomaterial–intestinal microbiota–intestinal epithelium interactions require further studies. Interestingly, some studies are appearing where the combination effects of nanomaterials and food ingredients are being explored, not only on the fate and physicochemical properties of the nanomaterials but also in terms of synergistic effects on the intestinal epithelium. There are indications that nanomaterials can induce, or interfere with, immune responses, but presently limited data are available. This topic closely correlates to the influence of nanomaterials on diseased states (e.g., inflamed intestine). In order to further investigate this there is a clear need for sophisticated (disease) models.

Together, the studies carried out to date on nanomaterials suggest that GIT epithelia may be prone to damage that is not always readily modeled *in vitro*, at least without the relatively laborious and expensive use of fully differentiated GIT cell models. However, the relative paucity of data regarding toxicity mechanisms and considerable uncertainty regarding realistic exposure levels and behavior of nanomaterials in the complex GIT environment make it challenging so far to predict the likely health effects of food‐borne nanomaterials.
